# Modifying the Autism Spectrum Rating Scale (6–18 years) to a Chinese Context: An Exploratory Factor Analysis

**DOI:** 10.1007/s12264-017-0104-7

**Published:** 2017-02-25

**Authors:** Hao Zhou, Lili Zhang, Xuerong Luo, Lijie Wu, Xiaobing Zou, Kun Xia, Yimin Wang, Xiu Xu, Xiaoling Ge, Yong-Hui Jiang, Eric Fombonne, Weili Yan, Yi Wang

**Affiliations:** 1 Department of Neurology, Children’s Hospital of Fudan University, Shanghai, 201102 China; 2 Department of Psychiatry, The Second Xiangya Hospital of Central South University, Changsha, 410008 China; 3 School of Public Health, Harbin Medical University, Harbin, 150081 China; 4 Child Development Center, The Third Affiliated Hospital, Sun Yat-Sen University, Guangzhou, 510000 China; 5 State Key Laboratory of Medical Genetics, Changsha, 400078 China; 6 Department of Child Healthcare, Children’s Hospital of Fudan University, Shanghai, 201102 China; 7 Children’s Hospital of Fudan University, Shanghai, 201102 China; 8 Division of Medical Genetics, Department of Pediatrics and Neurobiology, Duke University School of Medicine, Durham, NC 27710 USA; 9 Institute on Development and Disability, Oregon Health and Science University, Portland, OR 97239 USA; 10 Department of Clinical Epidemiology, Children’s Hospital of Fudan University, Shanghai, 201102 China; 11 Pediatric Department of Guizhou Provincial People’s Hospital, Guiyang, 550002 China

**Keywords:** Autism spectrum disorder, Screening, Epidemiology, Exploratory factor analysis, Children

## Abstract

The purpose of this study was to explore the psychometric properties of the Chinese version of the autism spectrum rating scale (ASRS). We recruited 1,625 community-based children and 211 autism spectrum disorder (ASD) cases from 4 sites, and the parents of all participants completed the Chinese version of the ASRS. A robust weighted least squares means and variance adjusted estimator was used for exploratory factor analysis. The 3-factor structure included 59 items suitable for the current sample. The item reliability for the modified Chinese version of the ASRS (MC-ASRS) was excellent. Moreover, with 60 as the cut-off point, receiver operating characteristic analysis showed that the MC-ASRS had excellent discriminate validity, comparable to that of the unmodified Chinese version (UC-ASRS), with area under the curve values of 0.952 (95% CI: 0.936–0.967) and 0.948 (95% CI: 0.930–0.965), respectively. Meanwhile, the confirm factor analysis revealed that MC-ASRS had a better construct validity than UC-ASRS based on the above factor solution in another children sample. In conclusion, the MC-ASRS shows better efficacy in epidemiological screening for ASD in Chinese children.

**Electronic supplementary material:**

The online version of this article (doi:10.1007/s12264-017-0104-7) contains supplementary material, which is available to authorized users.

## Introduction

Autism spectrum disorder (ASD) is a group of heterogeneous neurodevelopmental disorders characterized by deficits in social interaction and reciprocal communication, as well as restricted and repetitive interests and behaviors [1]. ASD has become a major worldwide issue in public health because its prevalence has significantly increased in many countries over the last few decades [2–4]. However, the causes of the progressive increase in the prevalence of ASD are not entirely clear. Potential contributing factors are changes in diagnostic criteria, increased attention within the medical community, and greater awareness among parents [5]. The etiology of autistic conditions nevertheless remains poorly understood, and the prevalence rate has varied substantially between studies and over time [6, 7].

ASD is an important cause of childhood disability worldwide. The prevalence of disability caused by autism is 2.38 per 10,000 individuals between 0 and 17 years old and 6.39 per 10,000 individuals between 4 and 6 years in the Chinese population [8]. However, at the national level, the prevalence of ASD in children in mainland China remains unknown. The usual approach to conducting a nation-wide epidemiological investigation of ASD in the Chinese general pediatric population (6–12 years) is to screen a representative population in order to identify children suspected of having ASD and to conduct next-step clinical assessments with systematic methods in order to obtain an accurate estimate of prevalence. A questionnaire-based epidemiologic study is an easy and efficient method of screening for ASD in the general population because it is easy to carry out and relatively inexpensive. However, the use of questionnaires relies on each participant’s understanding of the instructions for each individual item, which may vary according to the cultural context in different samples [9, 10]. Factor analysis has been widely used to investigate the latent structure of ASD questionnaires in different populations in cross-cultural environments [11, 12].

Most studies of the factor structure of ASD questionnaires have used Western populations. To date, Chinese versions of several screening tools for autism have been developed [13]; however, only a few studies have conducted factor analysis of these assessment tools. One study used samples of school-aged students recruited from primary school and participants from clinical settings to explore the Social Responsiveness Scale in a Chinese population [14]. The results supported a 4-factor structure for the Chinese version of this scale. Gau *et al.* conducted factor analysis and revealed a 3-factor structure for a social communication questionnaire in Chinese children [15]. Another study examined the Autism Spectrum Quotient, which involved 5 factors in the general Chinese population [16]. However, these studies were all based on populations in the Taiwan region. So far, only one study has conducted a factor analysis of a screening tool for ASD in the Chinese population in China’s mainland. Specifically, Sun *et al.* conducted a factor analysis of the Mandarin Chinese version of the Childhood Autism Spectrum Test in normal children and cases of autism; the results revealed a two-factor solution [17].

The Autism Spectrum Rating Scale (ASRS) is an ASD screening instrument developed by Goldstein and Naglieri [18]. It is available for two age ranges: 2–5 and 6–18 years. It is a newly-developed screening tool, and the only factor analysis of the ASRS has been conducted in a US population [18]. In a previous study, we demonstrated that the Chinese version of the ASRS is a useful instrument for screening autism in Chinese children. However, the construct validity of this version did not achieve the optimal value, with all values of the model fit <0.9 [19]. Therefore, to explore whether factor analysis of the ASRS in a sample of Chinese children is necessary, we measured the latent structure of the Chinese version (6–18 years old) and assessed the modified version in a different cultural environment, before its application in a national ASD screening program for children aged 6–12 years.

## Materials and Methods

### Participants

The samples were from a pilot national epidemiological study of ASD in Chinese school-aged children, conducted from January to July, 2014. To ensure data quality and that the sample was representative, participants were recruited from four cities geographically representative of China with a well-established base for epidemiological research: Shanghai, Harbin, Guangzhou, and Changsha.

The participants comprised two subsamples: (1) a community-based sample drawn from the parents of 2,053 children aged 6–12 years in Shanghai, Harbin, Guangzhou, and Changsha; and (2) a clinical sample of the parents of 211 individuals with autism. The children with ASD were recruited from the outpatients of participating institutions (The Children’s Hospital of Fudan University, Shanghai; The Third Affiliated Hospital of Sun Yat-Sen University, Guangzhou; The Second Xiangya Hospital of Central South University, Changsha; and Harbin Medical University, Harbin). All children with ASD had a clinical diagnosis made by a pediatrician according to the criteria of the Diagnostic and Statistical Manual of Mental Disorders, Fifth Edition.

### Screening Instrument

The ASRS was introduced to China using standard questionnaire translation procedures with the approval of Multi-Health Systems [20], and a previous study confirmed that the method is reliable [19]. The ASRS includes screening, Diagnostic and Statistical Manual of Mental Disorders, 4th Edition, Text Revision (DSM-IV-TR), and treatment scales, with a total of 71 items. In factor analyses, a 3-factor solution was most commonly found with the ASRS in western population. Three-factors comprising 60 items of the total 71 were generated for screening: Social/Communication (SC, 19 items), Unusual Behaviors (UB, 24 items), and Self-Regulation (SR, 17 items). These 3 scales were combined into a single composite score, the T-score, which was developed for screening purposes. The DSM-IV-TR scale contained 34 items based on expert experience from the total of 71 items, and a high score indicates that the child has a higher chance of being diagnosed as autistic by a psychiatrist. Finally, the treatment scale had a total of 69 out of the 71 items and included 8 subscales based on expert experience, which are: Peers Socialization (PS, 9 items), Adult Socialization (AS, 6 items), Social/Emotional Reciprocity (SER, 13items), Atypical Language (AL, 6 items), Stereotypy (ST, 5 items), Behavioral Rigidity (BR, 8 items), Sensory Sensitivity (SS, 6 items), and Attention (AT, 11 items). This can be used for ongoing monitoring of the clinical status of children with ASD.

### Study Procedure

The parents who gave written consent were invited to complete the Chinese version of the ASRS. Each parent was given a booklet that contained an information sheet, questionnaire, consent form, and guidance notes. Contact information for the research team was provided along with the scale in case parents had questions about the forms. The program was approved by the Ethics Review Board of the Children’s Hospital of Fudan University ([2012] No. 185).

### Statistics

The Chinese version of the ASRS was distributed to the parents of all eligible children in a pilot study. In all, 369 questionnaires were not returned, and 59 lacked basic information (e.g., name and date of birth). Another 160 questionnaires from the community-based sample had missing items: 126 (7.5%) had <5 missing items, and 34 (2%) had ≥5. In addition, 24 questionnaires from the clinical sample had missing items: 18 (8.5%) had <5, and 6 (2.8%) had ≥5. In total, 1,465 questionnaires from the community-based sample and 187 from the clinical sample were available for analysis.

The raw scores were used for factor analysis. We used the statistical package MPlus version 7.0 (Muthén & Muthén, Los Angeles, CA) to test the factor structure with exploratory factor analysis (EFA) [21]. Items in the ASRS were measured with 5-point Likert scales, and the variables were categorical. EFA was conducted with Geomin (oblique) rotation, which is a proper method for extracting categorical variables in factor analysis. The factor structure of the Chinese version of the ASRS was estimated using a robust weighted least squares means and variance-adjusted estimator [22]. This approach is considered to be more accurate for exploring the latent structure of questionnaires by identifying the factor structure of categorical variables than other methods based on continuous variables [23]. The χ^2^ goodness-of-fit test, the root mean square error of approximation (RMSEA), the comparative fit index (CFI), the Tucker–Lewis index (TLI), and the standardized root mean square residual (SMSR) were used to estimate the factor structure [24].

We selected the number of factors to retain via the Kaiser criterion, where components with eigenvalues >1, and the scree test, where components with eigenvalues before the ‘elbow’ of a scree plot, were retained [25]. The literature indicates that using both approaches is more accurate at identifying the correct number of factors than using only one method. In particular, the number of factors to retain can be overestimated if one method is used.

We considered factor loadings ≥0.3 to be outstanding, and an item was removed from further analysis if it had a factor loading <0.3 or cross-loading <0.1 [18]. In addition, to ensure that each factor was well measured, factors with <3 items were removed.

We used the standard ASRS T-score to conduct further analyses. Cronbach’s alpha was used to test item reliability [26], and receiver operating characteristic (ROC) curves were used to assess the performance of the questionnaire, as ROC analysis is a helpful method for determining the validity of questionnaires [27]. Specifically, we used ROC analysis to measure the discriminate validity of the Chinese version of the ASRS and computed the area under the curve (AUC) and 95% confidence intervals (CI). Ultimately, the sensitivity and specificity of the Chinese version of the ASRS for screening for ASD were analyzed.

## Results

### Demographic Characteristics of the Samples

A total of 1,465 questionnaires from the community-based sample and 187 questionnaires from the clinical sample were included in the analysis. The community-based sample included 752 boys (51.3%) with a mean age of 8.8 ± 1.8 years, and the clinical sample included 161 boys (86.1%) with a mean age of 8.9 ± 1.9 years. Those excluded were missing basic information and data necessary for statistical analysis. Statistics regarding age, sex, and site distribution are shown in Tables 1 and 2.

**Table 1:** Age and sex distribution of the reference sample.

Age	Community-based sample (*n* = 1465)			Clinical sample (*n* = 187)		
	Male *n* (%)	Female *n* (%)	Total	Male *n* (%)	Female *n* (%)	Total
6	125 (54.6)	104 (45.4)	229	44 (89.8)	5 (10.2)	49
7	123 (50.8)	119 (49.1)	242	28 (87.5)	4 (12.5)	32
8	134 (55.6)	107 (44.4)	241	18 (81.8)	4 (18.2)	22
9	118 (47.0)	133 (53.0)	251	25 (92.6)	2 (7.4)	27
10	99 (48.3)	106 (52.7)	205	17 (85.0)	3 (15.0)	20
11	106 (52.7)	95 (48.3)	201	17 (77.3)	5 (22.7)	22
12	47 (49.0)	49 (51.0)	96	12 (80.0)	3 (20.0)	15
Total	752 (51.3)	713 (48.7)	1465	161 (86.1)	26 (13.9)	187
*χ* ^*2*^	5.76			3.82		
*P*	0.45			0.70

**Table 2:** Site distribution of the reference sample.

City	Community-based sample (*n* = 1465)			Clinical sample (*n* = 187)		
	Male *n* (%)	Female *n* (%)	Total	Male *n* (%)	Female *n* (%)	Total
Shanghai	183 (49.7)	185 (50.3)	368	47 (85.5)	8 (14.5)	55
Guangzhou	221 (52.1)	203 (47.9)	424	40 (87.0)	6 (13.0)	46
Changsha	166 (50.0)	166 (50.0)	332	40 (85.1)	7 (14.9)	47
Harbin	182 (53.4)	159 (46.6)	341	34 (87.2)	5 (12.8)	39
Total	752 (51.3)	713 (48.7)	1465	161 (86.1)	26 (13.9)	187
χ^2^	1.29			0.12		
*P*	0.73			0.99		

The mean scores for the ASRS by type of sample and by gender are summarized in Table 3. The total score and SC, UB, and SR sub-scores were higher in the clinical sample than in the community-based sample. These significant differences still existed between the two samples for males and females (all *P* < 0.001).

**Table 3:** Mean ASRS scores by sample type and gender.

	Community-based sample (*n* = 1465)			Clinical sample (*n* = 187)			*P* ^*#*^
	All	Boys (*n* = 747)	Girls	All	Boys (*n* = 166)	Girls	
Total score	54.82 ± 7.0	55.78 ± 6.82	53.82 ± 7.04	69.23 ± 6.30	69.0 ± 6.47	71.48 ± 4.2	<0.011
SC	56.50 ± 9.44	57.36 ± 9.42	55.61 ± 9.40	74.49 ± 8.14	74.27 ± 8.45	76.20 ± 4.88	<0.011
SR	47.37 ± 8.36	48.71 ± 8.24	57.75 ± 6.40	59.90 ± 7.00	59.54 ± 8.23	65.62 ± 4.68	<0.011
UB	58.22 ± 6.31	58.69 ± 6.18	45.97 ± 8.26	64.84 ± 6.0	64.75 ± 6.11	62.76 ± 4.05	<0.011

^#^ *t* test results for comparisons of means between community-based and clinical samples. All *P* < 0.001 for all community children *versus* all children with ASD; all community boys *vs* all boys with ASD; all community girls vs all girls with ASD. *SC* Social Communication, *UB* Unusual Behavior, *SR* Self Regulation.

### Exploratory Factor Analysis

**Fig. 1:**
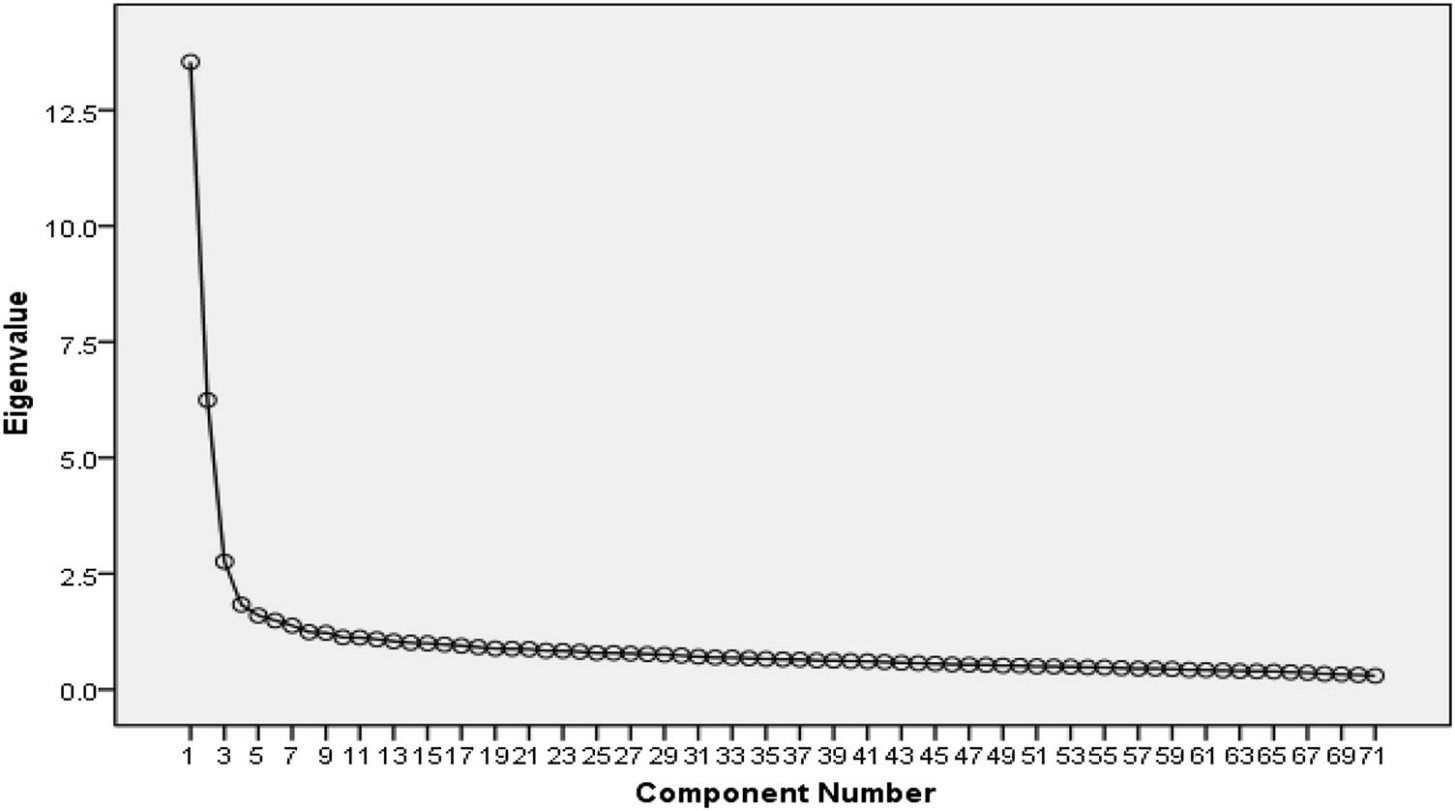
Screen plot.

The results of the EFA revealed thirteen factors with eigenvalues >1. While a break was apparent in the slope of plotted eigenvalues, the shape of the curve suggested that three factors were appropriate for the present sample (Fig. 1). Therefore, our model fit statistics were based on a three-factor structure, and each factor was extracted (Table 4).

**Table 4:** Model fit statistics by factor solutions from the exploratory factor analysis (71 items).

Factors	χ^2^			RMSEA	CFI	TLI	SMSR	Eigenvalues
	*χ* ^*2*^	*df*	*P*					
1	23546.570	2414	0.000	0.077	0.669	0.659	0.101	17.102
2	10707.564	2344	0.000	0.049	0.869	0.861	0.053	6.827
3	7348.148	2275	0.000	0.039	0.921	0.913	0.040	3.037

Index criteria for a model of good fit: RMSEA < 0.05, CFI > 0.90, TLI > 0.90, SMSR < 0.08. *CFI* comparative fit index, *RMSEA* root mean square error of approximation, *SMSR* standardized root mean square residual, *TLI* Tucker–Lewis index.

Among the 71 items, 12 were excluded: items 2 (becomes bothered by some fabrics or tags in clothes), 3 (seeks the company of other children), 4 (shows little emotion), 7 (has problems waiting his/her turn), 11 (avoids looking at people who speak to him/her), 14 (has trouble talking with other children), 26 (repeats or echoes what others have said), 34 (avoids looking at adults when a problem occurs), 46 (flaps his/her hands when excited), 52 (has problems paying attention to fun tasks), 59 (has trouble talking with adults), and 68 (reverses pronouns [e.g., you for me]). Items 2, 3, 4, 11, 46, and 52 were excluded because their factor loading was <0.30. Item 7 had a cross-loading on factors 2 (0.300) and 3 (0.232); item 14 had a cross-loading on factors 1 (0.376) and 3 (0.463); item 26 had a cross-loading on factors 2 (0.281) and 3 (0.304); item 34 had a cross-loading on factors 2 (0.327) and 3 (0.271); item 59 had a cross-loading on factors 1 (0.378) and 3 (0.463); and item 68 had a cross-loading on factors 1 (0.289) and 3 (0.342). Thus, these items were excluded as well.

Factor 1, “SC”, included 21 items (5, 8, 9, 10, 12, 15, 23, 28, 31, 32, 33, 39, 42, 43, 45, 47, 55, 56, 61, 69, and 70); factor 2, “SR”, included 14 items (1, 6, 16, 17, 27, 30, 35, 36, 37, 44, 57, 58, 60, and 71), and factor 3, “UB”, included 24 items (13, 18, 19, 20, 21, 22, 24, 25, 29, 38, 40, 41, 48, 49, 50, 51, 53, 54, 62, 63, 64, 65, 66, and 67). Thus, 59 items were retained for further analysis and the EFA was performed again on them. The model remained stable and met the criteria for the goodness-of-fit indices (RMSEA = 0.041, CFI = 0.926, TLI = 0.950, SRMR = 0.045). The item loadings for each factor of the Chinese version of the ASRS are shown in Table S1.

We conducted a confirm factor analysis based on the above factor solution in another population of normal children. The sample came from a primary school in the Minhang District of Shanghai: 671 children aged 6–12 years. The results revealed that this modified Chinese version (MC-ASRS) had a better construct validity than the unmodified version (UC-ASRS) [28].

### Item Reliability of the Chinese Version of the ASRS

We used the Cronbach’s alpha to test the item reliability [29]. The item reliability for the 59 items was 0.926 for the MC-ASRS and 0.915 for the UC-ASRS. Moreover, for the SC, SR, and UB subscales, Cronbach’s alpha was 0.908, 0.873, and 0.857 for the MC-ASRS and 0.87, 0.863, and 0.846, for the UC-ASRS, respectively. These results indicated that, regarding the item structure, the MC-ASRS had relatively better reliability (for the three subscales and total scores) than the UC-ASRS for the Chinese population (Table 5).

**Table 5:** Comparison of Cronbach’s alpha for each factor and the total score between the UC-ASRS and the MC-ASRS.

Factors	UC-ASRS	Cronbach’s alpha	MC-ASRS	Cronbach’s alpha
SC	19	0.87	21	0.908
SR	17	0.863	14	0.873
UB	24	0.846	24	0.857
Total	60	0.915	59	0.929

*MC-ASRS* modified Chinese version of the Autism Spectrum Rating Scale, *UC-ASRS* unmodified Chinese version of the scale, *SC* Social Communication, *SR* Self Regulation, *UB* Unusual Behavior.

### Optimal Cut-Offs of the Chinese Version of the ASRS

A previous study suggested that the ROC curve is a reliable method to determine the ideal cut-offs for questionnaires in psychiatric research on children [30]. Using an approach to determine the optimal sensitivity and specificity, we found that the conventionally-used cut-off of 60 (mean + 1 SD) for the MC-ASRS achieved a sensitivity of 94.2% and a specificity of 82.0%, in the current sample. The original study developing the ASRS suggested using a cut-off of 60 for the USA version [18]. Thus, we used a cut-off of 60 to compute the sensitivity and specificity of the UC-ASRS. The results (sensitivity 94.7%, specificity 77%) showed that, compared with the MC-ASRS, the UC-ASRS had a relatively equal sensitivity and a slightly lower specificity.

### Discriminate Validity of the Chinese Version of the ASRS

**Fig. 2:**
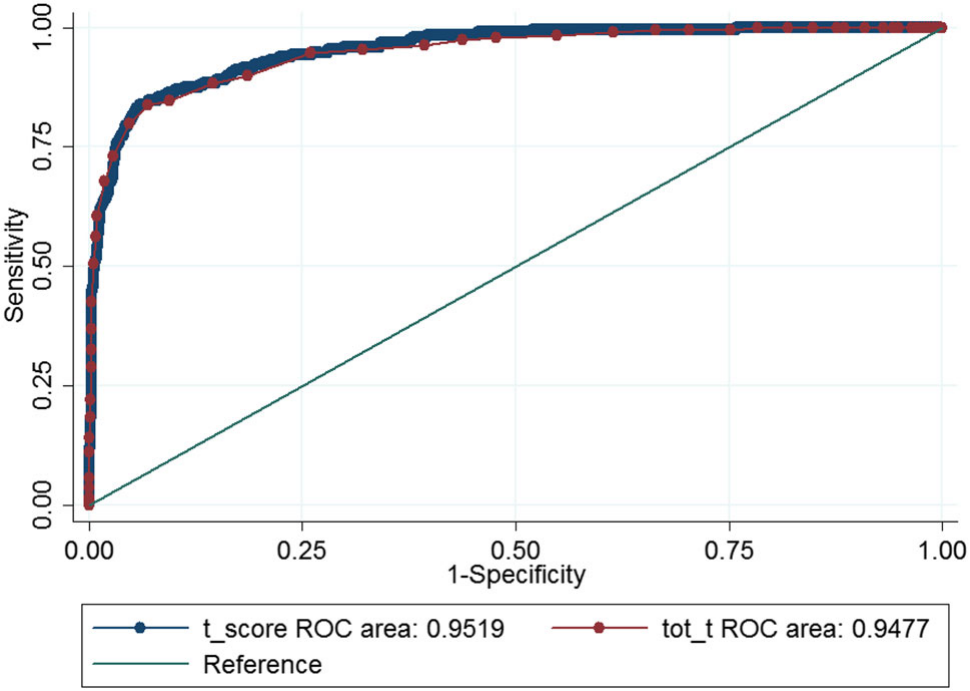
Receiver Operating Characteristic (ROC) curves for the total score for the MC-ASRS and UC-ASRS. MC-ASRS, modified Chinese version of the Autism Spectrum Rating Scale; UC-ASRS, unmodified Chinese version of the ASRS; t_score, total score of the MC-ASRS, tot_t, total score of the UC-ASRS.

We performed ROC analysis to test the overall discriminate validity of both the MC-ASRS and the UC-ASRS (Fig. 2). Using the same cut-off of 60, we found that both versions yielded AUCs > 0.9, with an AUC of the total score of 0.952 (95% CI: 0.936–0.967) for the MC-ASRS and 0.948 (95% CI: 0.930–0.965) for the UC-ASRS, indicating equally excellent discriminate validity for screening children with ASD. We performed further analysis separately on each gender and found that the scales performed even better among girls: AUC = 0.991; 95% CI: 0.980–1.000 for the MC-ASRS and 0.996; 95% CI: 0.991–1.000 for the UC-ASRS (Figs. S1 and S2).

## Discussion

A standard approach to determining the efficacy of an assessment tool is to determine whether scores on the scale are significantly higher for a clinical sample than for the general population. This indicates that the tool is able to easily identify cases in the general population. The ASRS is a newly-developed screening tool, and prior research has demonstrated that scores on its subscales are significantly higher in children with ASD than in normal children in the US population [18]. The current study demonstrates the efficacy of ASRS based on a Chinese sample.

EFA revealed the underlying structure of the MC-ASRS, which consisted of three domains related to the quality of ASD screening in the present sample. An EFA of the ASRS suggested that a 3-factor solution, comprising 60 of the total 71 items, was suitable for screening in western population. However, the MC-ASRS retained 59 items loaded on a comparable 3-factor structure. Moreover, the content of the 3 factors was similar to that of those in the original US version [18]. The only difference was that a change in the numbers of items contained in each factor was justified for the Chinese sample. The content of each factor may have differed between the MC-ASRS and the UC-ASRS for two reasons.

First, some items shifted from one factor to another in the MC-ASRS compared with the UC-ASRS. Second, in the MC-ASRS, some items in the UC-ASRS were removed, and other items from the 71-item total were added. These adjustments may have been justified because of cultural differences that may have affected the understanding of each concept. For instance, items 3 “will seek the company of other children” and 4 “shows little emotion” were removed from the MC-ASRS. In Western culture, a child exhibiting such behaviors may be considered to lack social skills, and his or her parents might think that the child is introverted and shy; in Chinese culture, however, such behavior is considered normal. The differences between the two versions were very similar for the SR and UB subscales, which may be attributed to different understanding of the same concepts between cultures, especially since the concepts of SR and UB are easy to confuse in Chinese culture. Thus, the shifting of many items between the UB and SR subscales is understandable. Expert judgments were required in the factor analysis when items shifted from one factor to another, which may have influenced the results. Our expert team thought that the MC-ASRS would be more suitable for a Chinese cultural environment. Previous studies have also demonstrated that cross-cultural influences may affect the factor structure of a questionnaire and that modifying questionnaires for different cultural backgrounds may be important [31, 32].

The EFA identified 12 items as potential candidates for deletion because of poor factor loadings in the MC-ASRS. Experts have suggested that as many items in the questionnaire as possible should be retained in a factor analysis. In this study, we deleted 12 items. The need to delete so many may be associated with the design of the ASRS questionnaire. Many well-informed autism scales have been designed mainly for screening. Initially, Dr. Sam Goldstein developed the ASRS not only for screening but also for diagnosis and monitoring the treatment of children with ASD. Therefore, the ASRS contains more items than other screening instruments for ASD. The UC-ASRS retained 60 items in the ASRS screening scale via EFA [18]. However, item assignment to the DSM-IV-TR and treatment scales was based on the content of the items, clinical experience, and the judgment of experts.

The analysis of item reliability demonstrated that Cronbach’s alpha for each factor and the total score was slightly better for the MC-ASRS than for the UC-ASRS. The cross-cultural environment is known to affect the performance of a questionnaire [33]. The high AUC values in the ROC analysis indicated that the discriminate validity of the MC-ASRS was strong and as high as that of the UC-ASRS in the Chinese reference sample. The results revealed that the MC-ASRS had excellent item reliability and discriminate validity and that the MC-ASRS had equal sensitivity and better specificity than the UC-ASRS. The confirm factor analysis based on the factor solution in another population of normal children [28] also demonstrated that the MC-ASRS had a better construct validity than the UC-ASRS, supporting its use as a reliable screening tool for ASD in children and adolescent populations in China.

## Limitations

The samples in our study were drawn from 4 cities. Differences in culture, language, and diversity are the most probable causes of the disparities in factor structure between the MC-ASRS and the UC-ASRS. Using EFA, we were unable to explore the specific contributions of each of these types of difference. As currently the EFA of the ASRS is conducted only in the US population, a comparison between the present results and those of other studies with respect to these issues cannot be made.

It is important to note that caution should be exercised in interpreting our results. Owing to missing data, the final analysis did not include all of the collected questionnaires, but the vast majority were included; thus, the exclusion of these ASRS questionnaires is unlikely to have affected the results of the EFA. The criteria used to determine salient loadings, the factor extraction and rotation methods, the methods of analysis, and the criteria used for indices of model fit may have affected the factor structure. However, we conducted EFA with reference to previous research methods [34].

## Conclusion

This is the first multisite study to use both community-based and clinical samples to test the MC-ASRS with EFA. The 3-factor solution of the MC-ASRS was stable and reliable, and it showed excellent discriminate validity, as well as good sensitivity and specificity. Our results thus demonstrated that the MC-ASRS is a useful and reliable tool for screening for the symptoms of autism in Chinese children.

## Electronic supplementary material

Below is the link to the electronic supplementary material.

Supplementary material 1 (PDF 85 kb)
