# Supplementary material for: Modifying the Autism Spectrum Rating Scale (6–18 years) to a Chinese Context: An Exploratory Factor Analysis
Source: Neurosci Bull. 2017 Feb 25;33(2):175–82. doi: 10.1007/s12264-017-0104-7 (PMC5360853; doi:10.1007/s12264-017-0104-7)
Supplement: Supplementary file 1 — Supplementary material 1 (PDF 85 kb) [file 12264_2017_104_MOESM1_ESM.pdf]

Electronic Supplemental Material

Table S1 Item loadings for the three factors of the Chinese version of the ASRS from the exploratory factor analysis

Items	SC	SR	UB
Item1	0.178	**0.548**	0.050
Item2	-0.054	0.166	0.182
Item3	0.222	-0.220	-0.046
Item4	0.137	0.084	0.200
Item5	**0.572**	0.066	0.012
Item6	0.022	**0.447**	0.056
Item7	0.225	0.300	0.232
Item8	**0.664**	-0.133	0.120
Item9	**0.579**	0.077	0.078
Item10	**0.513**	0.306	-0.104
Item11	0.257	0.244	0.267
Item12	**0.605**	0.046	0.079
Item13	-0.089	0.183	**0.392**
Item14	0.376	0.040	0.463
Item15	**0.641**	0.184	-0.020
Item16	0.143	**0.444**	0.190
Item17	0.089	**0.389**	0.281
Item18	0.386	-0.033	**0.492**
Item19	0.342	-0.012	**0.452**
Item20	0.207	0.212	**0.484**
Item21	0.160	0.274	**0.467**
Item22	-0.066	0.097	**0.576**
Item23	**0.642**	0.076	0.016
Item24	-0.283	0.016	**0.440**
Item25	0.075	-0.037	**0.521**
Item26	-0.184	0.281	0.304
Item27	0.026	**0.367**	0.247
Item28	**0.594**	0.293	-0.061
Item29	0.007	0.105	**0.413**
Item30	0.007	**0.760**	0.004
Item31	**0.630**	-0.180	0.235
Item32	**0.484**	0.174	-0.145
Item33	**0.652**	0.014	0.051

Item34	0.053	0.327	0.271
Item35	0.111	**0.710**	0.011
Item36	-0.084	**0.716**	-0.099
Item37	-0.143	**0.453**	0.241
Item38	0.232	-0.115	**0.470**
Item39	**0.606**	0.239	-0.079
Item40	-0.107	-0.050	**0.588**
Item41	0.030	0.227	**0.346**
Item42	**0.700**	-0.069	0.132
Item43	**0.603**	0.032	-0.099
Item44	0.194	**0.510**	0.010
Item45	**0.657**	0.028	-0.038
Item46	-0.356	0.227	0.204
Item47	**0.644**	0.199	-0.029
Item48	-0.081	0.082	**0.557**
Item49	-0.252	0.119	**0.514**
Item50	-0.010	0.207	**0.477**
Item51	-0.454	-0.195	**0.306**
Item52	0.264	0.235	0.237
Item53	0.018	0.264	**0.455**
Item54	-0.027	0.143	**0.449**
Item55	**0.620**	0.041	0.091
Item56	**0.597**	-0.135	0.227
Item57	0.139	**0.544**	0.023
Item58	-0.043	**0.499**	0.235
Item59	0.378	0.114	0.463
Item60	-0.038	**0.569**	0.175
Item61	**0.564**	-0.013	0.054
Item62	-0.056	0.130	**0.501**
Item63	0.017	0.234	**0.484**
Item64	0.134	0.063	**0.454**
Item65	0.030	0.056	**0.484**
Item66	0.415	-0.043	**0.575**
Item67	-0.046	0.260	**0.386**
Item68	0.289	0.182	0.342
Item69	**0.673**	0.057	0.097
Item70	**0.711**	-0.127	0.126
Item71	0.165	**0.535**	0.149

The values in bold are salient loadings

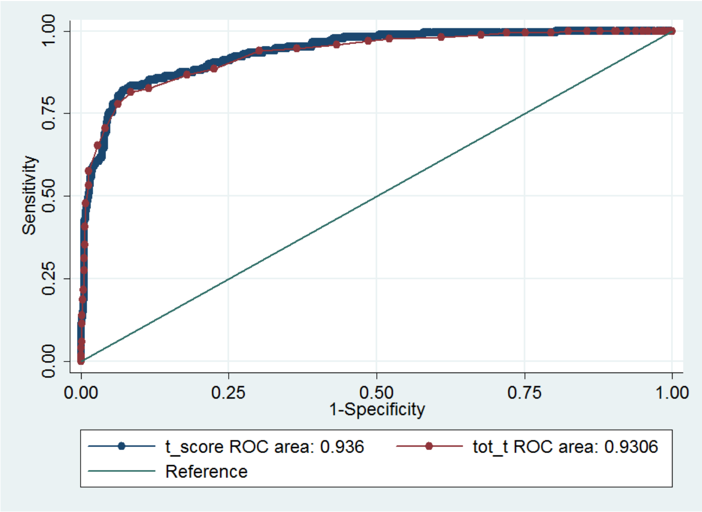

Fig. S1 Receiver Operating Characteristic (ROC) curves for the total score for the MC-ASRS and the UC-ASRS for males. Note: MC-ASRS, modified Chinese version of the Autism Spectrum Rating Scale; UC-ASRS unmodified Chinese version of the Autism Spectrum Rating Scale; t_score, total score of the MC-ASRS, tot_score, total score of the UC-ASRS.

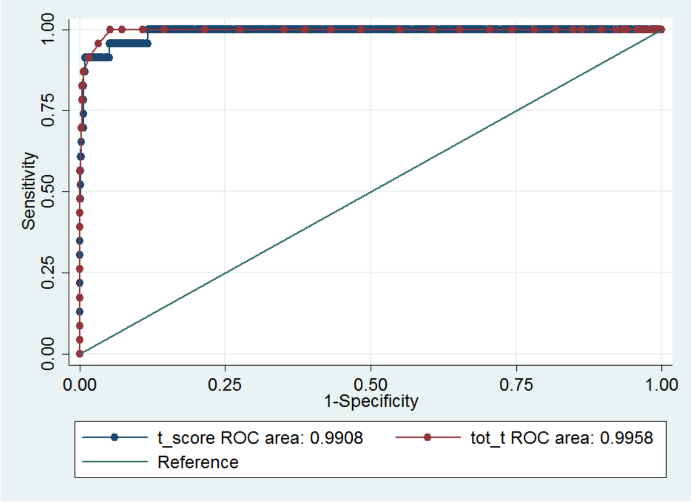

Fig. S2 Receiver Operating Characteristic (ROC) curves for the total score for the MC-ASRS and the UC-ASRS for females. Note: MC-ASRS, modified Chinese version of the Autism Spectrum Rating Scale; UC-ASRS unmodified Chinese version of the Autism Spectrum Rating Scale; t_score, total score of the MC-ASRS, tot_score, total score of the UC-ASRS
